# Patient experiences in ulcerative colitis: conceptual model and review of patient-reported outcome measures

**DOI:** 10.1007/s11136-024-03612-4

**Published:** 2024-03-05

**Authors:** Chong Kim, Fiona L. Brown, Caroline Burk, Milena Anatchkova, Nashmel Sargalo, Ankita Kaushik

**Affiliations:** 1 HEOR, Gilead Sciences, Foster City, CA USA; 2 Patient-Centered Research, Evidera, London, UK; 3 HEOR Consultant, Laguna Beach, CA USA; 4 Patient-Centered Research, Evidera, Bethesda, MD USA

**Keywords:** Clinical outcome assessment, Conceptual model, Gap analysis, Inflammatory bowel disease, Patient-reported outcome measure, UC-PRO-Signs and Symptoms, Ulcerative colitis

## Abstract

**Purpose:**

To identify symptoms and their impacts on daily functioning and health-related quality of life (HRQoL) experienced by adult patients with ulcerative colitis (UC) and evaluate patient-reported outcome (PRO) measures for UC clinical studies.

**Methods:**

A conceptual model of symptoms and impacts of UC were developed from a literature review. PRO measures were identified from the literature, clinical trials databases, health technology assessment submissions, and regulatory label claims, and were selected for conceptual analysis based on disease specificity and use across information sources. PRO measures covering the most concepts when mapped against the conceptual model were assessed for gaps in psychometric properties using Food and Drug Administration (FDA) guidance and consensus-based standards for the selection of health measurement instruments (COSMIN) criteria.

**Results:**

The conceptual model grouped the 52 symptom concepts and 72 proximal and distal impacts into eight, two, and five dimensions, respectively. Of 65 PRO measures identified, eight underwent conceptual analysis. Measures covering the most concepts and assessed for psychometric properties were the Inflammatory Bowel Disease Questionnaire, Symptoms and Impacts Questionnaire for UC, UC-PRO symptoms modules, UC-PRO impact modules, and Crohn’s and UC Questionnaire; all had good or excellent support for content validity. The UC-PRO Signs and Symptoms fully met FDA guidance and COSMIN criteria for content validity and most psychometric properties.

**Conclusion:**

Existing PRO measures assess concepts relevant to patients with UC, but all PRO measures reviewed require further psychometric evaluation to demonstrate they are fit for purpose.

**Supplementary Information:**

The online version contains supplementary material available at 10.1007/s11136-024-03612-4.

## Plain English summary

Patients with ulcerative colitis (UC) experience symptoms that can be disabling. New treatment options are needed for patients with UC. Patient-reported outcome (PRO) measures are questionnaires that ask patients about disease-related symptoms and the disease impacts on their lives. Regulators (such as the US Food and Drug Administration [FDA]) recommend that clinical trials of new treatments for UC should include PRO measures to assess treatment effects. In this study, we aimed to gain an understanding of the symptoms and impacts experienced by patients with UC and to evaluate available PRO measures for use in UC clinical trials. We searched the published literature and identified 52 symptoms and 72 impacts related to the disease experience of patients with UC. From these, we developed a model of UC. The most common UC-related symptoms were diarrhea, incontinence/leaking/lack of bowel control, urgency, rectal bleeding, frequent bowel movements, fatigue, and abdominal pain. The most common UC-related impacts were anxiety, depression, and inability to conduct daily activities. We identified and evaluated several PRO measures for use in clinical trials that assess symptoms and impacts relevant to patients with UC. None of the PRO measures identified included all symptoms and impacts relevant to patients with UC. The PRO measures identified need to undergo further testing.

## Introduction

Inflammatory bowel disease (IBD) is a chronic and debilitating immune-mediated condition affecting more than 6.8 million people worldwide [1]. The prevalence of IBD has increased in recent decades, with a corresponding increase in years of life lived with IBD-related disability [1]. UC is a form of IBD involving chronic inflammation of the colonic mucosa, including the rectum, and is characterized by periods of relapsing and remitting disease activity [2]. Patients with UC experience symptoms that are intrusive and can be disabling, including bloody diarrhea, bowel urgency, frequent bowel movements, and abdominal pain [2, 3].

The clinical goals of UC treatment are focused on inducing and maintaining remission, response, mucosal healing, and restoring health-related quality of life (HRQoL) [2, 4]. Although several classes of therapeutic agents are available for UC, a marked proportion of patients either do not respond or lose response to therapy [5–8]. New treatment options are needed for patients with UC and several are in clinical development [9]. In its draft guidance for industry, the US Food and Drug Administration (FDA) recommends that clinical trials assessing new UC treatments should assess clinical remission and response as primary and secondary endpoints, respectively [3]. The definitions of clinical remission and response in the draft guidance are based on the modified Mayo Score (mMS); a composite endpoint comprising subscores for stool frequency, rectal bleeding, and centrally read endoscopy [3].

Additionally, when evaluating the effects of investigational drugs, the FDA encourages sponsors to assess symptoms of UC that are not captured by the mMS but have been identified by patients as important by using fit-for-purpose PRO measures [3]. PRO measures are the best clinical outcome assessment (COA) type to capture patients’ experiences [10]. The European Medicines Agency (EMA) guideline on the development of new treatments for UC and FDA guidance on patient-focused drug development (PFDD) highlights the importance of capturing patient-reported symptoms and their impacts on daily functioning and HRQoL [10, 11]. The EMA guideline recommends further development and validation of PRO measures for use as primary outcome parameters in clinical trials in UC, while noting that validated HRQoL measures may be reported as secondary endpoints [11]. As part of its PFDD guidance, the FDA recommends searching the literature, repositories, and other resources for existing COAs that measure the concepts of interest in the relevant context of use [10]. A conceptual model can be used to assess if an available COA fully captures the concepts of interest [10].

This study aimed to identify symptoms and impacts experienced by adult patients with UC, develop a conceptual model based on concepts important to these patients, and evaluate available PRO measures for appropriateness for inclusion in UC clinical studies.

## Methods

### Study methodology overview

**Fig. 1:**
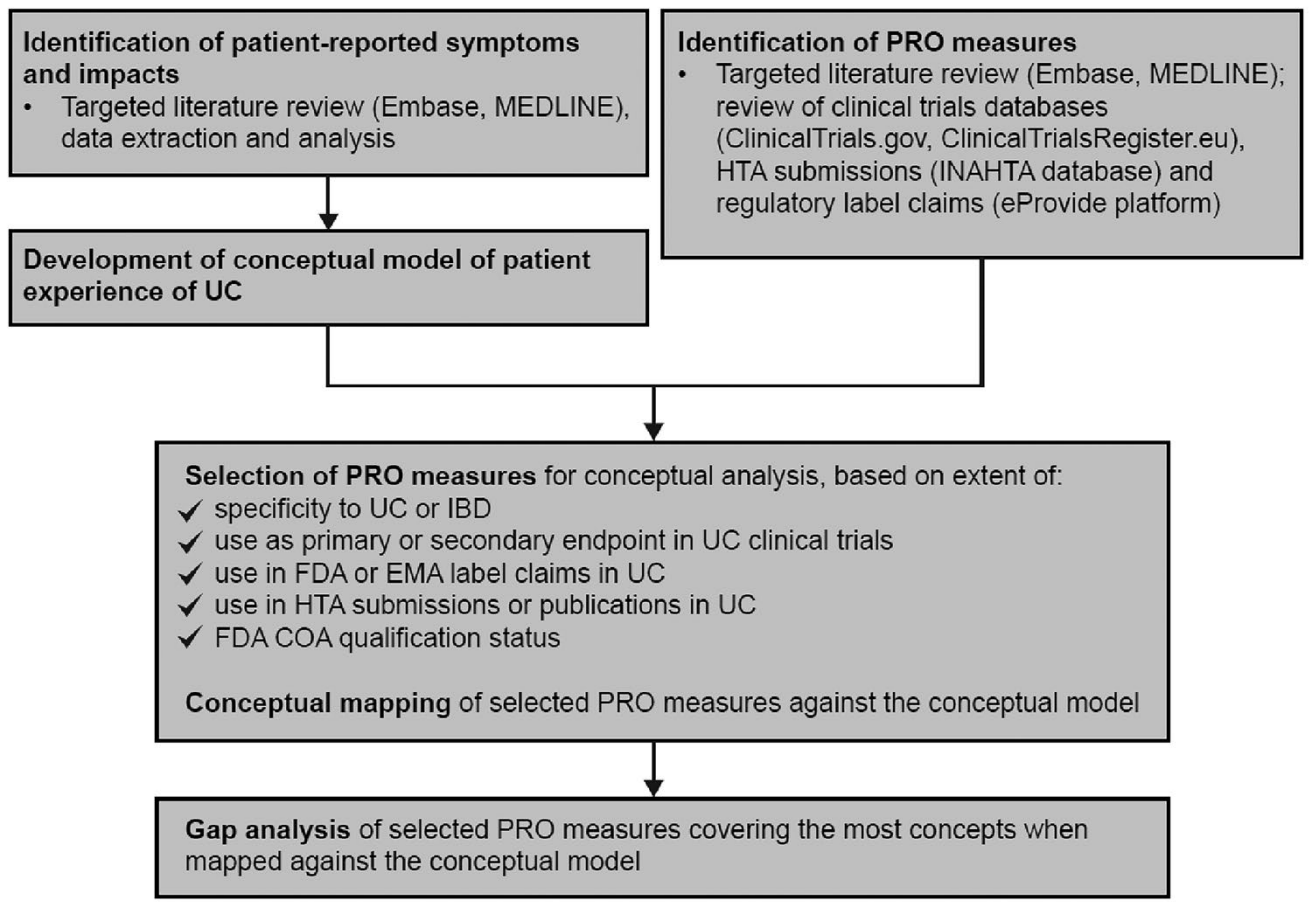
Overview study methodology. *COA* clinical outcome assessment, *EMA* European Medicines Agency, *FDA* Food and Drug Administration, *HTA* health technology assessment, *IBD* inflammatory bowel disease, *INAHTA* International Network of Agencies for Health Technology Assessment, *PRO* patient-reported outcome, *UC* ulcerative colitis

An overview of the study methodology is shown in Fig. 1. A conceptual model of the adult patient experience of UC was developed based on patient-reported symptoms and impacts identified from a targeted literature review (TLR). A subset of PRO measures used in UC clinical studies, identified using a wide range of resources, was chosen based on specified criteria and mapped against the conceptual model. Selected PRO measures (*n* = 5) were further evaluated using gap analysis for appropriateness for inclusion in UC clinical studies.

### Identification of patient-reported symptoms and impacts

A targeted literature search (TLR1) was conducted in Embase and Medline using the Ovid platform to identify studies reporting symptoms and impacts experienced by adult patients with UC (see Supplemental Table S1 for search strategy). The search period start date was January 1, 2016 for full-text articles and January 1, 2020 for congress abstracts. English-language publications published up to November 23, 2021 (the date of TLR1) were eligible for review. Publications were screened for eligibility using predefined inclusion criteria based on population, intervention/comparator, outcomes, and study design (PICOS) (see Supplemental Table S2 for details): qualitative and quantitative publications were selected if they provided information on symptoms of UC in adult patients (≥ 18 years or reported as “adults” in publication) and their impacts on patients’ daily functioning and HRQoL. Publications were excluded if they reported exclusively on Crohn’s disease, did not report results separately for UC, consisted of patients younger than 18 years, consisted of symptoms and impacts that were not patient reported, were case reports, or if the full-text/congress poster was not available. Data on patient characteristics and symptoms and impacts of UC were extracted from the selected publications. Impacts were categorized as “proximal” if they were directly related to UC and “distal” if they occurred as a result of a proximal impact.

### Conceptual model development

A model conceptualizing patient-reported experiences of UC was developed based on the symptoms and impacts data extracted from TLR1. The model was inspired by Wilson and Cleary’s 1995 model in which HRQoL was presented in terms of functioning and overall quality of life as proximal and distal impacts, respectively [12]. Thematic analysis was used to group health concepts by common themes into dimensions capturing symptoms, and proximal and distal impacts of UC [12, 13], based on the descriptions reported in the literature.

### Identification of PRO measures

To identify studies reporting PRO measures, including HRQoL measures, for use in clinical trials in UC and/or IBD, a second targeted literature review (TLR2) was conducted in Embase and Medline using Ovid (see Supplemental Tables S3 and S4 for search strategies). Full-text, English-language articles were eligible for review. Publications were screened and selected for eligibility using predefined PICOS inclusion criteria (see Supplemental Table S5 for details).

The following additional resources were searched: US Clinical Studies Database (ClinicalTrials.gov) and European Union (EU) Clinical Trials Register (ClinicalTrialsRegister.eu), to identify PRO measures used as primary or secondary endpoints in UC; International Network of Agencies for Health Technology Assessment (INAHTA), for PRO measures used in UC in relation to health technology assessment (HTA) submissions; PRO product label database of new molecular entities approved by the FDA or the EMA (eProvide), for products with a PRO claim in UC; and the FDA website (www.fda.gov), for PRO measures under FDA qualification for use in UC.

Searches to identify PRO measures were conducted on November 23 and 24, 2021. The search period start date was January 1, 2019 for all searches except TLR2 and the INAHTA database search, for which the start date was January 1, 2009 (the publication year of the FDA guidance on use of PRO measures in medical product development to support labeling claims [14]). Data on identified PRO measures were extracted from the resources.

### Conceptual analysis and mapping

PRO measures were selected for conceptual analysis based on the extent that they satisfied the following criteria: disease specific to UC or IBD, evidence of use as a primary or secondary endpoint in a clinical trial in UC, evidence of use in FDA or EMA label claims, evidence of use in HTA submissions or publications, and/or FDA qualification status. Concepts evaluated by the selected PRO measures were mapped against the conceptual model.

### Gap analysis

PRO measures that covered the most concepts when mapped to the conceptual model were selected for gap analysis. The selected measures were evaluated for their psychometric properties using FDA guidance and consensus-based standards for the selection of health measurement instruments (COSMIN) criteria [14–18]. The following criteria were used to review each PRO measure: conceptual framework, item and scale refinement, reliability (internal consistency, test–retest reliability, inter-/intra-rater reliability), validity (content validity, construct validity), ability to detect change, responder analysis, interpretation of scores, and linguistic equivalence of translations. Using these criteria, the properties of each PRO measure were assessed and rated as either “fully meets criteria,” “partially meets criteria,” “significant concerns about meeting criteria,” or “no evidence that process, method or test was completed,” with the latter two ratings indicating gaps in evidence.

## Results

### Patient-reported symptoms and impacts

TLR1 identified 18 publications for data extraction (see Supplementary Figure S1 for flow chart and the reference list for publication details) [19–36]. The publications described the experiences of patients from a range of geographical regions including Australia, Canada, Finland, France, Germany, Italy, Japan, Malta, Mexico, the Netherlands, New Zealand, Norway, Singapore, South Korea, Spain, Sweden, the UK, and the USA.

Data extraction identified 52 symptoms and 72 impacts of UC. The most frequently reported symptoms (mentioned in ≥ 4 publications) were diarrhea, incontinence/leaking/lack of bowel control, urgency, rectal bleeding, frequent bowel movements, fatigue, abdominal pain, sleep disturbance, flatulence, pain, tiredness, weight loss, and colectomy resulting as a complication of UC-related symptoms. The most frequently reported impacts (mentioned in ≥ 4 publications) were anxiety, depression, inability to conduct daily activities, embarrassment, affected relationships with others, worry/fear, inability to travel, having to plan around UC/prepare for incontinence, and absence from work. Based on the literature review findings, 29 impacts were categorized as proximal to UC (i.e., directly related to UC) and 43 as distal to UC (i.e., occurring as a result of a proximal impact).

### Conceptual model

**Fig. 2:**
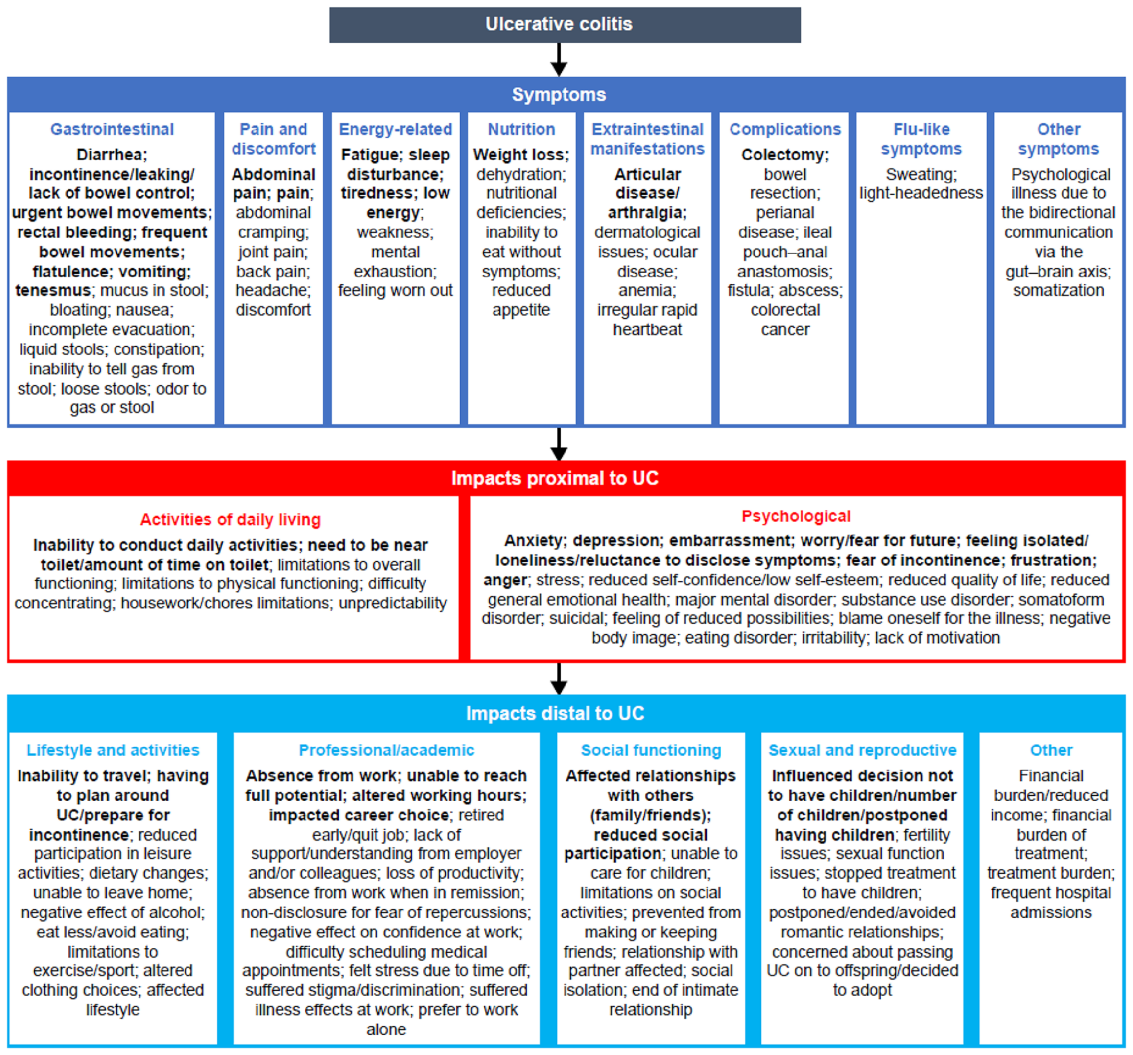
Conceptual model of UC. Concepts described in the literature were characterized as symptoms of UC (e.g., “diarrhea,” “pain”), proximal impacts of UC if these were reported as being a direct result of UC symptoms (e.g., “need to be near toilet/amount of time on toilet”), and distal impacts of UC if these were downstream impacts of UC symptoms that affect how a patient feels or functions (e.g., “inability to travel”). Bold font indicates most frequently reported concepts in three or more publications. *UC* ulcerative colitis

A conceptual model of the patient experience of UC was developed (Fig. 2; see Supplementary Tables S6–S8 for symptom, proximal, and distal impact concepts, dimensions, and associated publications). The conceptual model grouped the 52 symptom concepts into eight dimensions (gastrointestinal, pain and discomfort, energy related, nutrition, extraintestinal manifestations, complications, flu-like symptoms, and other). The model grouped the 29 impacts proximal to UC into two dimensions (activities of daily living and psychological) and the 43 impacts distal to UC into five dimensions (lifestyle and activities, professional/academic, social functioning, sexual and reproductive, and other).

### PRO measures

Taken together, 65 PRO measures were identified from the TLR, clinical trial databases, HTA, and label review, of which 19 were specific to UC or IBD (Table 1).

**Table 1:** UC and/or IBD-specific PRO measures identified from reviewed sources

	Reviewed sources			
	TLRs	Clinical trials databases	HTA submissions	FDA or EMA label claims
Bristol Stool Scale		✓		
CUCQ	✓		✓^a^	
CUCQ+			✓^a^	
DUCS	✓			
ePRO bowel urgency	✓	✓		
IBD-CTRL	✓	✓^a^		
IBD-DI	✓	✓		
IBD-Disk		✓		
IBDQ	✓	✓^a^	✓	✓
IBDSI	✓			
Mayo subscores (stool frequency, rectal bleeding)	✓	✓		
MIAH	✓			
PRO-2		✓		
SIBDQ	✓	✓	✓^a^	✓
SIQ-UC	✓	✓		
UC-PRO				
Symptom modules (UC-PRO/SS^b^, UC-PRO systemic symptoms)	✓	✓		
Impact modules (UC-PRO-DC, UC-PRO-DLI, UC-PRO-EI)		✓		
Urgency NRS		✓		
WPAI-UC	✓	✓	✓	✓

*CUCQ* Crohn’s and Ulcerative Colitis Questionnaire, *CUCQ*+ CUCQ with post-colectomy extension, *DUCS* Daily Ulcerative Colitis Signs and Symptoms, *EMA* European Medicines Agency, *ePRO* electronic PRO, *FDA* US Food and Drug Administration, *HTA* health technology assessment, *IBD* inflammatory bowel disease, *IBD-CTRL* Inflammatory Bowel Disease Control, *IBD-DI* Inflammatory Bowel Disease-Disability Index, *IBD-Disk* Inflammatory Bowel Disease Disk, *IBDQ* Inflammatory Bowel Disease Questionnaire, *IBDSI* Inflammatory Bowel Disease Symptom Inventory, *MIAH* Monitor Inflammatory Bowel Disease at Home, *NRS* numerical rating scale, *PRO* patient-reported outcome, *SIBDQ* Short Inflammatory Bowel Disease Questionnaire, *TLR* targeted literature review, *SIQ-UC* Symptoms and Impacts Questionnaire for UC, *UC* ulcerative colitis, *UC-PRO-DC* UC-PRO-Daily Coping, *UC-PRO-DLI* UC-PRO-Daily Life Impact, *UC-PRO-EI*, UC-PRO-Emotional Impact, *UC-PRO/SS* UC-PRO Signs and Symptoms, *WPAI-UC* Work Productivity and Activity Impairment-ulcerative colitis

^a^Used as primary endpoint

^b^Submitted for FDA qualification and has EMA endorsement as UC clinical trial endpoint

TLR2 identified 11 publications on PRO measures for data extraction (see Supplementary Figure S2 for flow chart) [37–47]. The publications predominantly described patients from the USA and Canada. The most frequently reported PRO measures were the Short Inflammatory Bowel Disease Questionnaire (SIBDQ), Inflammatory Bowel Disease Questionnaire (IBDQ), and Inflammatory Bowel Disease-Disability Index (IBD-DI), which are disease specific and the 36-item Short Form Health Survey (SF-36) and EuroQol five-dimensions questionnaire (EQ-5D), which are generic.

Of clinical trials examined in the US and EU clinical trial databases, 62% (115/185) did not report a PRO endpoint. A PRO measure was listed as a primary or secondary endpoint in 59 trials in UC. Two PRO measures were listed as primary endpoints: the Inflammatory Bowel Disease Control (IBD-CTRL) was registered as a primary endpoint in ClinicalTrialsRegister.eu, and the IBDQ was listed as a primary endpoint in both ClinicalTrials.gov and ClinicalTrialsRegister.eu.

The review of HTA publication and submission records (20 reviewed; seven with data extraction) identified 11 PRO measures. Three of the PRO measures were listed as primary endpoints: the SIBDQ, Crohn’s and UC Questionnaire (CUCQ), and CUCQ with post-colectomy extension (CUCQ+).

The FDA and EMA labels review identified 15 medications for UC with a COA claim that incorporated seven PRO measures in total. Only EMA-approved labels contained PRO evidence from patients with UC. Two measures for use in UC were submitted to the FDA as part of the COA qualification program: the UC-PRO Signs and Symptoms (UC-PRO/SS; qualification status: in legacy process [under review prior to the passage of the 21st Century Cures Act]) and the TUMMY-UC (qualification process: withdrawn) [48, 49]. The EMA provided a letter of support for use of the UC-PRO/SS as an endpoint in clinical trials with patients with UC [50].

### Conceptual analysis and mapping

PRO measures specific to UC or IBD were selected for conceptual analysis based on the extent to which they met the specified selection criteria (Fig. 1), and were mapped to the conceptual model. These were the IBDQ; SIBDQ; Work Productivity and Activity Impairment-ulcerative colitis questionnaire (WPAI-UC); Symptoms and Impacts Questionnaire for UC (SIQ-UC); UC-PRO symptoms modules (UC-PRO/SS; UC-PRO-Systemic Symptoms); UC-PRO impacts modules (UC-PRO-Daily Coping [UC-PRO-DC], UC-PRO-Daily Life Impact [UC-PRO-DLI], and UC-PRO-Emotional Impact [UC-PRO-EI]); CUCQ; and PRO-2. UC-PRO symptoms modules were considered separately from the impacts modules for the conceptual analysis and mapping.

Table 2 lists the number of concepts covered by each of the selected PRO measures for the conceptual model symptom, proximal impact, and distal impact concepts, with a total for all concepts covered included for ease of comparison across measures. The IBDQ provided the most comprehensive coverage overall, accounting for 36 of the 124 concepts that were identified through TLR1 and included in the conceptual model. Taken together, the UC-PRO modules were second, covering 34 of the identified concepts; separately, the symptoms modules accounted for 14 concepts and the impacts modules for 20 concepts. The CUCQ included 32 concepts and the SIQ-UC included 29 concepts. Considering symptoms specifically, the SIQ-UC was the most comprehensive and included 18 of the 52 symptom concepts identified. The 29 proximal impacts were most comprehensively covered by the IBDQ, which accounted for 12 of the proximal impact concepts. For the 43 distal impacts, three measures (IBDQ, UC-PRO impacts modules, and CUCQ) provided equal concept coverage, with each including eight concepts.

**Table 2:** Conceptual mapping of selected PRO measures against the conceptual model for UC

Number of CM concepts covered in each PRO	IBDQ	SIBDQ	WPAI-UC	SIQ-UC	UC-PRO modules		CUCQ	PRO-2
					UC-PRO/SS; UC-PRO-Systemic Symptoms	UC-PRO-DC; UC-PRO-DLI; UC-PRO-EI		
CM symptom concepts (*n* = 52)	16	7	0	18	14	1	14	2
CM proximal impact concepts (*n* = 29)	12	4	2	5	0	11	10	0
CM distal impact concepts (*n* = 43)	8	3	5	6	0	8	8	0
All CM concepts (*n* = 124)	36	14	7	29	14	20	32	2
					UC-PRO modules total: 34			

*CM* conceptual model, *CUCQ* Crohn’s and Ulcerative Colitis Questionnaire, *IBDQ* Inflammatory Bowel Disease Questionnaire, *PRO* patient-reported outcome, *SIBDQ* Short Inflammatory Bowel Disease Questionnaire, *SIQ-UC* Symptoms and Impacts Questionnaire for UC, *UC* ulcerative colitis, *UC-PRO-DC* UC-PRO-Daily Coping, *UC-PRO-DLI* UC-PRO-Daily Life Impact, *UC-PRO-EI* UC-PRO-Emotional Impact, *UC-PRO/SS* UC-PRO Signs and Symptoms, *WPAI-UC* Work Productivity and Activity Impairment-UC

### Gap analysis

The five PRO measures covering the most concepts when mapped to the conceptual model were included for gap analysis of their content validity and psychometric properties to evaluate whether the measures can be considered fit for purpose (Table 3) [25, 41, 51–71]. Table 4 summarizes the properties of the PRO measures included in the gap analysis. These were the IBDQ, SIQ-UC, UC-PRO/SS, UC-PRO-impacts modules, and CUCQ. All five measures were developed for use in UC or IBD and have undergone psychometric evaluation.

**Table 3:** Gap analysis of selected PRO measures covering the most concepts when mapped against the conceptual model

	IBDQ	SIQ-UC	UC-PRO/SS	UC-PRO-DC; UC-PRO-DLI; UC-PRO-EI	CUCQ
Conceptual framework	+++	+++	+++	+++^a^	+++
Item and scale refinement	++	++	+++	+++	++
Reliability					
Internal consistency	+++	ø	+++	+++	++
Test–retest	+++	ø	+++	+++	++
Inter- and intra-rater	++^b^	−	−	−	−
Validity					
Content	+++	+++	+++	++	+++
Known groups	++	ø	+++	+++	ø
Convergent, discriminant	++	ø	+++	+++	+++
Factor analysis	ø	ø	+++	+++	ø
Ability to detect change over time	++	ø	+++	ø^a^	+++
Responder analysis	ø	ø	+++	ø^a^	ø
Interpretation of score	ø	ø	++	ø^a^	ø
Linguistic equivalence	+++	ø	+++^c^	+++^c^	ø

Assessment using FDA guidance and COSMIN criteria [14–17]: +++, fully meets criteria; ++, partially meets criteria; +, significant concerns about meeting criteria; ø, no evidence that process, method or test was completed; −, not relevant

*CUCQ* Crohn’s and Ulcerative Colitis Questionnaire, *COSMIN* consensus-based standards for the selection of health measurement instruments, *FDA* US Food and Drug Administration, *IBDQ* Inflammatory Bowel Disease Questionnaire, *PRO* patient-reported outcome, *SIQ-UC* Symptoms and Impacts Questionnaire for UC, *UC* ulcerative colitis, *UC-PRO-DC* UC-PRO-Daily Coping, *UC-PRO-DLI* UC-PRO-Daily Life Impact, *UC-PRO-EI* UC-PRO-Emotional Impact, *UC-PRO/SS* UC-PRO Signs and Symptoms

^a^Manuscript in development

^b^Assessment of nurse-administered versus self-administered questionnaire performed.

^c^Certification that translations meet regulatory-recommended procedure with forward- and back-translation and cognitive debriefing provided

**Table 4:** Summary of properties of PRO measures included in gap analysis

	PRO measure				
	IBDQ	SIQ-UC	UC-PRO/SS^a^	UC-PRO-DC; UC-PRO-DLI; UC-PRO-EI^b^	CUCQ^c^
Objective	To assess QoL of patients with IBD	To assess IBD-related symptoms in patients with UC	To assess GI signs and symptoms of UC in clinical trials	To assess daily coping, daily life impacts and emotional impacts in patients with UC	To assess Crohn’s and UC-specific QoL
Domains	*Four Domains:* bowel symptoms; systemic symptoms; emotional functioning; social functioning	*Three Domains:* daily bowel movement report; daily symptom report; weekly impact assessment	*UC-PRO/SS*: *Two Scales:* bowel signs and symptoms; abdominal symptoms	*Three modules:* daily coping; daily life impact; emotional impact	*No formal domain structure but four principal components:* emotional symptoms; bowel symptoms; social activities; general symptoms
Number of items	32	29	*UC-PRO/SS*: 9; *UC-PRO-Systemic Symptoms*: 5	*UC-PRO-DC:* 6; *UC-PRO-DLI:* 9; *UC-PRO-EI:* 8	32
Recall period	Past 2 weeks	*Symptoms:* past 24 h; *Impacts:* past 7 days	Past 24 h	*UC-PRO-DC:* past 24 h; *UC-PRO-DLI and UC-PRO-EI:* past 7 days	Past 2 weeks
Response options	7-point Likert severity and frequency scales	Multiple response options including dichotomous yes/no answers, specific time and 5-point Likert frequency and severity scales	Combination of dichotomous yes/no responses and 4-point Likert scales	*UC-PRO-DC:* dichotomous yes/no answers; *UC-PRO-DLI:* 5-point Likert severity scales; *UC-PRO-EI:* 5-point Likert frequency scales	Combination of number of days during past 2 weeks that concept was experienced and 4-point Likert frequency scale
Scoring	Score range, total: 32–224 (Bowel symptoms: 10–70; Systemic symptoms: 5–35; Emotional function: 12–84; Social function: 5–35), with higher scores reflecting better QoL	To be determined: SIQ-UC validation is a secondary endpoint of EU Clinical Trial 2019–002485-12	No total score across scales. *Bowel Signs and Symptoms Scale:* scored as mean across all items; score range 0–7 for item 1 (number of bowel movements) and 0–4 for all other items, with lower scores indicating lesser symptomatic experience. *Abdominal Symptoms Scale*: scored as mean across all items; score range 0–4 for all items, with lower scores indicating lesser symptomatic experience	No total score across modules. *UC-PRO-DC*: based on average weekly score, with score range 0–6 daily/7 days; higher scores indicate more coping mechanisms employed. UC-PRO-DLI: mean across all items in scale, with score range 0–4 for each item; lower scores indicate lower daily living impacts experienced. *UC-PRO-EI:* mean across all items in scale, with score range 0–4 for each item; lower scores indicate lower emotional impacts experienced	Score range, total: 0–272, calculated by summing all valid responses and dividing by number of completed questions (calculated only if ≥ 24/32 questions answered). Higher scores indicating worse QoL. Questions with four responses scored as 0, 1, 2 or 3 in ascending severity; questions with responses between 0 and 14 days scored as actual value; questions with wording in the reverse direction scored reversed; questions with responses between 0 and 1 rescaled by dividing responses by maximum score (3 or 14)
Clinically meaningful change	Mean detectable change suggested to be statistically significant is between 0.6 and 1.0 per item	To be determined: SIQ-UC validation is a secondary endpoint of EU Clinical Trial 2019-002485-12	Minimal clinically important difference, bowel domain: 6 points; functional domain: 2 points [70] Minimum clinically meaningful change, bowel domain: 4.85–6.31; functional domain: 1.48–2.07 Responder definition, bowel domain: ≥ 5 points reduction; functional domain: ≥ 1.5 points reduction	NR	NR
Interpretation thresholds in relation to disease activity	NR	To be determined: SIQ-UC validation is a secondary endpoint of EU Clinical Trial 2019-002485-12	*Bowel Signs and Symptoms Scale* and *Abdominal Symptoms Scale*: mean scores significantly higher in patients with active disease than those judged to be in clinical remission	NR	NR
Conditions of use	License fee: approximately $5000 for master license and approximately $1000 per study site usually charged. Each study site signs site license agreement	Available for use in clinical trials. Information and license agreement to be provided on submission of request for use	License fee: $35,000 per study/use and $2000 for existing translation; fee is the same irrespective of how many modules are used. Covers use for duration of study and all study activities provided limitations and obligations outlined in agreement are complied with. Fee is waived if study sponsor pays for development of translations for new modules for that language		Freely available to use by healthcare professionals to support patient care without license fees

*CUCQ* Crohn’s and Ulcerative Colitis Questionnaire, *EU* European Union, *GI* gastrointestinal, *IBD* inflammatory bowel disease, *IBDQ* Inflammatory Bowel Disease Questionnaire, *NR* not reported, *PRO* patient-reported outcome, *QoL* quality of life, *SIQ-UC* Symptoms and Impacts Questionnaire for UC, *UC* ulcerative colitis, *UC-PRO-DC* UC-PRO-Daily Coping, *UC-PRO-DLI* UC-PRO-Daily Life Impact, *UC-PRO-EI* UC-PRO-Emotional Impact, *UC-PRO/SS* UC-PRO Signs and Symptoms

^a^The UC-PRO/SS is Module One of a suite of PRO measures assessing patient experience of UC. Its aim is to standardize the quantification of GI signs and symptoms of UC in clinical trials through direct report from patient ratings

^b^Each UC-PRO module is a suite of PRO measures assessing patient experience of UC

^c^The CUCQ and CUCQ with post-colectomy extension (CUCQ+) were developed based on the 32-item UK version of the IBDQ

The IBDQ is a 32-item quality of life assessment for patients with IBD [52, 55, 56, 64, 66–68]. Patients and healthcare providers were involved in the content development. Existing evidence supported good internal consistency reliability (Cronbach’s alpha ≥ 0.70), test–retest reliability (intraclass correlation coefficient [ICC] ≥ 0.66 [range: 0.66 to 0.99]), and linguistic equivalence. Limited evidence of item and scale refinement, known-groups validity, convergent/discriminant validity, and ability to detect change was identified as part of the gap analysis, indicating that these components only partially met FDA guidance and COSMIN criteria, and additional evidence is recommended. There was a gap in evidence available on factor analysis, responder analysis, or interpretation of scores for the IBDQ.

The SIQ-UC is a 29-item PRO measure created to evaluate symptoms and impacts in UC clinical trials to facilitate drug development [25]. Patients with UC were involved in content development. It was the only measure developed using endoscopic evidence [25]. Limited evidence of item and scale refinement were available in the literature, indicating a potential gap wherein available information only partially met the FDA guidance and COSMIN criteria. Evidence was missing in the literature for internal consistency reliability, test–retest reliability, known-groups validity, convergent/discriminant validity, factor analysis, responsiveness, responder analysis, interpretation of scores, and linguistic evidence, indicating significant gaps with this measure, owing to its novel nature. Psychometric validation is ongoing within clinical trials.

The UC-PRO/SS is a nine-item PRO measure developed for the assessment of gastrointestinal signs and symptoms in a clinical trial setting among patients with UC [41, 59–61, 70]. Patients with mild to severe UC were involved in content development. The UC-PRO/SS met FDA guidance and COSMIN criteria for all parameters included as part of the gap analysis, except for score interpretation, for which information on handling of missing data was not identified and which, thus, only partially met FDA and COSMIN standards. Existing evidence supported good internal consistency reliability (Cronbach’s alpha range: 0.66 to 0.80, depending on the domain assessed) and test–retest reliability (ICC range: 0.71 to 0.81). The measure was developed well, with documented evidence of a clear conceptual framework and both item and scale refinement. All constructs related to reliability and validity were evident and well documented.

The UC-PRO impacts modules (UC-PRO-DC, UC-PRO-DLI, and UC-PRO-EI) are scored separately [58, 60]. Limited evidence of content validity was available in the literature; thus, this construct partially meets FDA and COSMIN standards. Existing evidence supported good internal consistency (Cronbach’s alpha range across modules: 0.71 to 0.94) and test–retest reliability (ICC range: 0.84 to 0.93). Additionally, the measure fully met FDA and COSMIN standards for known-groups validity, convergent/discriminant validity, factor analysis, and linguistic equivalence. No evidence for measure responsiveness, responder analysis, or score interpretation was available in the literature, indicating a gap in the measure development.

The CUCQ is a 32-item PRO measure created from the UK version of the IBDQ to assess the disease-specific HRQoL of patients with IBD in clinical practice and in healthcare evaluation [51, 52, 55, 62, 67, 71]. Patients and two expert review panels were involved in content development. Limited existing evidence supported internal consistency reliability (Cronbach’s alpha range: 0.845 to 0.91) and test–retest reliability (ICC: 0.94). Evidence was good for convergent/discriminant validity and ability to detect change over time (responsiveness). There was an evidence gap in known-groups validity, factor analysis, responder analysis, interpretation of scores, and linguistic equivalence for the CUCQ.

Taken together, the gap analysis showed that the IBDQ, SIQ-UC, UC-PRO/SS, and CUCQ have excellent support for content validity (i.e., fully met FDA guidance and COSMIN criteria). The UC-PRO impacts modules have good support for content validity (i.e., partially met FDA guidance and COSMIN criteria). The IBDQ and the UC-PRO/SS had the most validation evidence of the measures included in the gap analysis. The UC-PRO/SS additionally meets FDA guidance and COSMIN criteria for most psychometric properties. All five measures had good evidence of acceptable patient burden, taking between 6 and 15 min to complete.

## Discussion

Knowing which symptoms and impacts are important to patients with UC, and which concepts and PRO measures are appropriate for capturing these symptoms and impacts, is crucial for a patient-focused approach when assessing new treatments for UC. Results from the literature review conducted as part of the current study showed that patients with UC experience a wide range of disease-related symptoms and impacts: 124 different symptoms and life impacts were identified. The most frequently noted symptoms gastrointestinal symptoms, pain, and fatigue. The most common impacts were on activities of daily living and the psychological well-being of patients.

The FDA and the EMA are placing an increased focus on including validated PRO measures in clinical studies [3, 11]. The FDA draft guidance (Guidance 3) on selecting, developing or modifying fit-for-purpose COAs was published after the current study was conducted [10]. Existing measures with evidence in the intended context of use are generally preferred [10]. Searches of scientific literature, repositories of measures, and other resources are recommended to identify existing COAs that measure concepts of interest [10]. A conceptual model is useful for representing patients’ experiences resulting from their disease, and can help sponsors assess if an available COA fully captures the concepts of interest [10]. The UC conceptual model that was developed in the current study grouped the identified UC symptom concepts into eight dimensions, impacts proximal to UC into two dimensions, and impacts distal to UC into five dimensions. Symptoms and proximal impacts were considered the most suitable to measure from a regulatory perspective when assessing UC and its treatment in clinical trials.

Patients are increasingly being integrated as stakeholders in drug development [10]. Including patient voices in UC clinical trials using validated PRO measures ensures that data on concepts important to patients are available to inform regulatory decision-making [14]. The current work identified 65 PRO measures that were used in clinical trials in patients with UC or IBD, of which 19 were specific to UC or IBD. Despite FDA guidance to support PFDD [10, 72, 73], issued under the 21st Century Cures Act, inclusion of PRO information in IBD product labels was found to be rare, and more likely to occur in EMA-approved than FDA-approved labels. Overall, only 38% of the clinical trials examined included a primary or secondary PRO-based endpoint in the study design. Only EMA-approved labels contained PRO evidence from patients with UC. However, following the completion of the review the FDA approved a label claim in UC based on eDairy PRO data evaluating UC-specific symptoms of bowel urgency and abdominal pain [74]. Due to timing of approval, these single item PROs were not included in the gap analysis.

Gap analysis of the five PRO measures that met the current study’s selection criteria and had the widest concept coverage when mapped against the conceptual model showed that all five of the measures had good or excellent support for content validity. The SIQ-UC was the only measure that included participants with endoscopic evidence of disease severity in its development process [25]. The IBDQ and UC-PRO/SS had the most validation evidence, and the UC-PRO/SS fully met FDA PRO guidance for content validity and most psychometric properties. Although there was an evidence gap on responder analysis for the IBDQ, a 16 to 32-point decrease in IBDQ score has been suggested as a clinically relevant improvement and a total score of at least 170 points as indicating remission based on clinical trials in Crohn’s disease, observational data, and expert opinion [64, 75]. These results indicate that relevant PRO measures, including HRQoL measures (IBDQ and CUCQ), exist for assessing symptoms and proximal impacts important to patients with UC in clinical trials, although none of the PRO measures comprehensively captures all relevant concepts important to patients identified in the literature reviewed. Based on available evidence, some psychometric evaluation would be required for all reviewed PRO measures. A combination of PRO measures may be useful for evaluating fully the range of relevant concepts. The potential burden to patients of completing more than one PRO measure should be considered; for the five measures included in the gap analysis, time to complete ranged from 6 to 15 min.

The COSMIN checklist was used to appraise methodological quality in two systematic reviews of IBD-specific HRQoL measures, published in 2015 and 2017 [52, 76]. Both reviews found the IBDQ to have the strongest published evidence of validity among identified measures. Our study expands on previous evaluations with the inclusion of additional and newer UC-specific measures (e.g., SIQ-UC, UC-PRO/SS, UC-PRO impact modules), and focusing on PRO use as UC trial endpoints, label claims, HTA submissions, and FDA qualification status. Historically, HRQoL measures were not favored by the FDA to support effectiveness claims (they were accepted by the EMA) [77, 78]. Thus, the regulatory focus of our study affected the selection of measures for analysis, with some HRQoL measures not being evaluated despite HRQoL being included as an outcome of interest in the TLRs and HRQoL concepts being included in the conceptual model.

This study has several key strengths. To our knowledge, it provides the most up-to-date literature analysis of PRO measures for use in UC clinical trials. A rigorous methodology was used, and a wide range of sources were reviewed. A limitation of this work is that literature reviews were targeted, not systematic. PRO measures were selected for conceptual analysis based on the extent to which they met the selection criteria; thus, measures with little or no evidence of use in UC clinical trials, regulatory label claims, HTA submissions or FDA COA qualification program status, based on our database searches, were not considered for further review. PRO measures can also have a role in clinical practice to help with patient–physician communication and disease management decision-making; however, the focus of the current work was on PROs for use in clinical trials.

## Conclusion

Fifty-two symptoms and 72 impacts of UC were identified. The most frequently reported symptoms were gastrointestinal symptoms, pain, and fatigue, and the most common impacts were on activities of daily living and the psychological well-being of patients. Existing PRO measures assess symptom and impact concepts relevant to patients with UC but do not fully capture all relevant concepts. Based on the results of the current study, a combination of PRO measures is suggested to capture relevant concepts of UC; for example, the IBDQ together with the UC-PRO/SS to capture UC symptoms, or the IBDQ together with the UC-PRO impact modules to capture proximal impacts. The selection of measures in future clinical trials would depend on the study design and treatment characteristics. All PRO measures reviewed require further psychometric evaluation to demonstrate that they are fit for purpose.

### Supplementary Information

Below is the link to the electronic supplementary material.

Supplementary file1 (DOCX 506 KB)

## Data Availability

All data generated or analyzed during this study are included in this published article and its supplementary information files.
