# Supplementary material for: Patient experiences in ulcerative colitis: conceptual model and review of patient-reported outcome measures
Source: Qual Life Res. 2024 Mar 5;33(5):1373–87. doi: 10.1007/s11136-024-03612-4 (PMC11045605; doi:10.1007/s11136-024-03612-4)
Supplement: Supplementary file 1 — Supplementary file1 (DOCX 506 KB) [file 11136_2024_3612_MOESM1_ESM.docx]

# Supplemental materials

**Supplemental Table S1**. Targeted literature search in Ovid (Embase and Medline) for studies reporting patient-reported symptoms and impacts of ulcerative colitis

| Search number | Search terms | Results |
| --- | --- | --- |
| 1 | exp ulcerative colitis/ or exp colitis, ulcerative/ or (ulcerative colitis or idiopathic proctocolitis or colitis gravis).ti,ab. | 138 603 |
| 2 | *symptomatology/ or (symptom$ or sign$ or impact or impacts).ti. | 2 107 432 |
| 3 | ((clinical or disease) adj3 manifest$).ti. | 26 748 |
| 4 | 1 and (2 or 3) | 5278 |
| 5 | Qualitative Research/ or Questionnaires/ or Focus Groups/ or questionnaire/ or attitude/ or (focus-group$ or discourse analysis or content analysis or ethnographic$ or constant comparative or qualitative or grounded-theory or narrative$).ti,ab. | 2 314 467 |
| 6 | ((Patient or participant or subject or caregiver or family or clinician or doctor$ or physician$ or practitioner$) adj3 (report$ or experience$ or assessment)).ti,ab. | 519 028 |
| 7 | *outcomes research/ or *"Outcome Assessment (Health Care)"/ or *outcome assessment/ or *Patient Reported Outcome Measures/ or *patient-reported outcome/ or *Interviews as Topic/ or *interview/ or *questionnaire/ or *"Surveys and Questionnaires"/ or *rating scale/ or (interview$ or questionnaire$ or instrument$ or scale$ or inventor$ or score$ or survey or tool or self-assess$ or self-complet$ or self-eval$ or reported-outcome or patient-rat$ or PROM or PREM or self-eval$ or patient-centered outcome$ or functional status or functional assessment or outcome measure$ or outcomes measure$ or outcome research or outcomes research or outcome assessment$ or outcomes assessment$ or hrql or hrqol or hr-qol or qol or patient-based outcome$).ti. or patient-reported.ti,ab. | 1 323 812 |
| 8 | 4 and (5 or 6 or 7) | 695 |
| 9 | 8 not (animals/ not humans/) | 695 |
| 10 | case reports/ or case study/ or case report$.jx. or case report$.jw. or (case report or case study or case series or woman or man or child or adolescent or female or male or boy or girl or infant or unusual case).ti. | 3 918 532 |
| 11 | (Ephemera or "Introductory Journal Article" or News or "Newspaper Article" or Editorial or Comment or Overall or Letter or Short Survey or Tombstone or Books).pt. or in vitro Techniques/ or in vitro study/ or (commentary or editorial or comment or letter or mice or rat or mouse or animal or murine).ti. | 8 724 155 |
| 12 | review.pt. not (systematic or meta$).mp. | 3 978 343 |
| 13 | 9 not (10 or 11 or 12) | 668 |
| 14 | limit 13 to (article or article in press) | 328 |
| 15 | limit 13 to (conference abstract and yr="2020-current") | 70 |
| 16 | 14 or 15 | 374 |
| 17 | limit 16 to English language | 364 |
| 18 | limit 17 to yr="2011-Current" | 217 |
| 19 | remove duplicates from 18 | 171 |

**Supplemental Table S2**. PICOS inclusion criteria for screening and selection of studies reporting patient-reported symptoms and impacts of ulcerative colitis

| **PICOS category** | **Inclusion criteria** |
| --- | --- |
| Population | Adult patients with ulcerative colitis |
| Intervention/comparator | No limits |
| Outcomes | Symptoms and impacts of ulcerative colitis   - Burden of disease - Functional impacts - Physical impacts - Psychological/emotional impacts - Social impacts - Behavioral impacts - Short- and long-term complications |
| Study design | Qualitative studies   - Qualitative interviews - Focus group discussions - Targeted/narrative literature reviews   Quantitative studies   - Cross-sectional survey studies - Prospective and retrospective cohort studies - Randomized controlled trials - Observational cohort studies - Epidemiological studies - Systematic reviews |

PICOS: population, intervention/comparator, outcomes, study design.

**Supplemental Table S3**. Targeted literature search in Ovid (Embase and Medline) for studies reporting PRO measures for use in clinical trials in ulcerative colitis

| Search number | Search terms | Results |
| --- | --- | --- |
| 1 | exp ulcerative colitis/ or exp colitis, ulcerative/ or (ulcerative colitis or idiopathic proctocolitis or colitis gravis).ti,ab. | 138 603 |
| 2 | (registry or registries).ti,ab. | 397 523 |
| 3 | 1 and 2 | 2 139 |
| 4 | ((Patient or participant or subject or caregiver or family) adj3 (report$ or experience$ or assessment)).ti. | 66 191 |
| 5 | exp *Interviews as Topic/ or exp *interview/ or exp *questionnaire/ or exp *"Surveys and Questionnaires"/ or exp *rating scale/ or (interview$ or questionnaire$ or instrument$ or scale$ or inventor$ or score$ or survey or tool).ti. | 1 275 584 |
| 6 | (self-assess$ or self-complet$ or self-eval$ or reported-outcome or patient-rat$ or patient-reported or PROM or PREM or self-eval$ or patient-centered outcome$ or functional status or functional assessment or outcome measure$ or outcomes measure$ or outcome research or outcomes research or outcome assessment$ or outcomes assessment$ or hrql or hrqol or hr-qol or qol).ti. or patient-based outcome$.ti,ab. | 79 571 |
| 7 | exp *outcomes research/ or exp *"Outcome Assessment (Health Care)"/ or exp *outcome assessment/ or exp *Patient Reported Outcome Measures/ or exp *patient-reported outcome/ or exp *functional status/ or exp *functional assessment/ | 122 371 |
| 8 | exp *"Quality of Life"/ or exp *quality of life/ or quality-of-life.ti. | 262 320 |
| 9 | or/4-8 | 1 678 588 |
| 10 | 3 and 9 | 107 |
| 11 | limit 10 to English language | 104 |
| 12 | limit 11 to yr="2009-Current" | 93 |
| 13 | 12 not ((exp animal/ or nonhuman/) not exp human/) | 93 |
| 14 | (case report or case series or woman or man or child or adolescent or female or male or boy or girl or infant).ti. or exp case report/ or exp case study/ or case report$.jn. or case report$.jx. | 5 977 970 |
| 15 | (Congress or Ephemera or "Meeting Abstract" or "Introductory Journal Article" or News or "Newspaper Article" or Biography or Editorial or Comment or Letter or Overall).pt. or (commentary or editorial or comment or mouse or mice or rat or rats or animal).ti. | 7 558 748 |
| 16 | in vitro study/ or In Vitro Techniques/ | 1 768 237 |
| 17 | 13 not (14 or 15 or 16) | 91 |
| 18 | limit 17 to (article or article in press) [Limit not valid in Ovid Medline, Ovid Medline Daily Update, Ovid Medline In-Process, Ovid Medline Publisher; records were retained] | 51 |
| 19 | remove duplicates from 18 | 39 |

**Supplemental Table S4**. Targeted literature search in Ovid (Embase and Medline) for studies reporting PRO measures for use in clinical trials in ulcerative colitis and/or inflammatory bowel disease

| Search number | Search terms | Results |
| --- | --- | --- |
| 1 | exp inflammatory bowel disease/ or crohn disease/ or ulcerative colitis/ or exp inflammatory bowel diseases/ or crohn disease/ or colitis, ulcerative/ or ((inflammatory adj (bowel or colon)) or ibd).ti,ab,kw. or (crohn* or (ulcerative colitis or idiopathic proctocolitis or colitis gravis)).ti,ab. or ((Microscopic or Collagenous or Lymphocytic) adj2 colitis).ti,ab,kw. or (pancolitis or rectitis or proctocolitis or procto-colitis or colorectitis or rectocolitis or recto-colitis or recto-sigmoiditis or rectosigmoiditis or procto-sigmoiditis or proctosigmoiditis or proctitis or ileocitis).ti,ab,kw. or ((terminal or regional) adj2 (ileitis or enteritis)).ti,ab,kw. or (granuloma* adj2 (colitis or enteritis)).ti,ab,kw. | 341 407 |
| 2 | (registry or registries).ti,ab. | 397 523 |
| 3 | 1 and 2 | 4 588 |
| 4 | ((Patient or participant or subject or caregiver or family) adj3 (report$ or experience$ or assessment)).ti. | 66 191 |
| 5 | exp *Interviews as Topic/ or exp *interview/ or exp *questionnaire/ or exp *"Surveys and Questionnaires"/ or exp *rating scale/ or (interview$ or questionnaire$ or instrument$ or scale$ or inventor$ or score$ or survey or tool).ti. | 1 275 584 |
| 6 | (self-assess$ or self-complet$ or self-eval$ or reported-outcome or patient-rat$ or patient-reported or PROM or PREM or self-eval$ or patient-centered outcome$ or functional status or functional assessment or outcome measure$ or outcomes measure$ or outcome research or outcomes research or outcome assessment$ or outcomes assessment$ or hrql or hrqol or hr-qol or qol).ti. or patient-based outcome$.ti,ab. | 79 571 |
| 7 | exp *outcomes research/ or exp *"Outcome Assessment (Health Care)"/ or exp *outcome assessment/ or exp *Patient Reported Outcome Measures/ or exp *patient-reported outcome/ or exp *functional status/ or exp *functional assessment/ | 122 371 |
| 8 | exp *"Quality of Life"/ or exp *quality of life/ or quality-of-life.ti. | 262 320 |
| 9 | or/4-8 | 1 678 588 |
| 10 | 3 and 9 | 243 |
| 11 | limit 10 to English language | 238 |
| 12 | limit 11 to yr="2009-Current" | 218 |
| 13 | 12 not ((exp animal/ or nonhuman/) not exp human/) | 218 |
| 14 | (case report or case series or woman or man or child or adolescent or female or male or boy or girl or infant).ti. or exp case report/ or exp case study/ or case report$.jn. or case report$.jx. | 5 977 970 |
| 15 | (Congress or Ephemera or "Meeting Abstract" or "Introductory Journal Article" or News or "Newspaper Article" or Biography or Editorial or Comment or Letter or Overall).pt. or (commentary or editorial or comment or mouse or mice or rat or rats or animal).ti. | 7 558 748 |
| 16 | in vitro study/ or In Vitro Techniques/ | 1 768 237 |
| 17 | 13 not (14 or 15 or 16) | 214 |
| 18 | limit 17 to (article or article in press) [Limit not valid in Ovid Medline, Ovid Medline Daily Update, Ovid Medline In-Process, Ovid Medline Publisher; records were retained] | 112 |
| 19 | remove duplicates from 18 | 85 |

PRO, patient-reported outcome.

**Supplemental Table S5**. PICOS inclusion criteria for screening and selection of studies reporting PRO measures for use in clinical trials in ulcerative colitis and/or inflammatory bowel disease

| **PICOS category** | **Inclusion criteria** |
| --- | --- |
| Population | Adult patients with ulcerative colitis or inflammatory bowel disease |
| Intervention/comparator | No limits |
| Outcomes | Self-assessment PRO measures of:   - Symptoms and impacts of ulcerative colitis   - Burden of disease   - Functional impacts   - Physical impacts   - Psychological/emotional impacts   - Social impacts   - Behavioral impacts - Short- and long-term complications - Health-related quality of life |
| Study design | Qualitative studies   - Qualitative interviews - Focus group discussions   Quantitative studies   - Cross-sectional and longitudinal validation studies - PRO measure development studies - Prospective and retrospective cohort studies - Randomized controlled trials - Observational cohort studies - Epidemiological studies - Systematic reviews |

PICOS, population, intervention/comparator, outcomes, study design; PRO, patient-reported outcome.

**Supplemental Table S6**. UC symptom concepts and dimensions

| Dimension | Symptom concept | Number of studies reporting concept | Citation |
| --- | --- | --- | --- |
| Gastrointestinal | Diarrhea | 9 | Byron 2020 [1]; Calvet 2018 [2]; Carpio 2016 [3]; Chan 2017 [4]; Dulai 2020 [5]; Garcia-Alanis 2021 [6]; Hashash 2018 [7]; Newton 2019 [8]; Rapport 2019 [9] |
|  | Incontinence/leaking/ lack of bowel control | 8 | Byron 2020 [1]; Carpio 2016 [3]; Dubinsky 2021 [10]; Dulai 2020 [5]; Garcia- Alanis 2021 [6]; Hibi 2020 [11]; Newton 2019 [8]; Vollebregt 2018 [12] |
|  | Urgent bowel movements | 8 | Byron 2020 [1]; Carpio 2016 [3]; Dubinsky 2021 [10]; Dulai 2020 [5]; Fourie 2018 [13]; Ghosh 2021 [14]; Hibi 2020 [11]; Rapport 2019 [9] |
|  | Rectal bleeding | 8 | Calvet 2018 [2]; Carpio 2016 [3]; Dubinsky 2021 [10]; Dulai 2020 [5]; Ghosh 2021 [14]; Hibi 2020 [11]; Newton 2019 [8]; Rapport 2019 [9] |
|  | Frequent bowel movements | 8 | Byron 2020 [1]; Calvet 2018 [2]; Dulai 2020 [5]; Fourie 2018 [13]; Ghosh 2021 [14]; Hibi 2020 [11]; Newton 2019 [8]; Rapport 2019 [9] |
|  | Flatulence | 5 | Calvet 2018 [2]; Carpio 2016 [3] Dubinsky 2021 [10]; Dulai 2020 [5]; Newton 2019 [8] |
|  | Vomiting | 3 | Byron 2020 [1]; Dulai 2020 [5]; Newton 2019 [8] |
|  | Tenesmus | 3 | Dulai 2020 [5]; Hibi 2020 [11]; Newton 2019 [8] |
|  | Mucus in stool | 2 | Dubinsky 2021 [10]; Dulai 2020 [5] |
|  | Bloating | 2 | Dulai 2020 [5]; Newton 2019 [8] |
|  | Nausea | 2 | Dulai 2020 [5]; Newton 2019 [8] |
|  | Incomplete evacuation | 2 | Hibi 2020 [11]; Newton 2019 [8] |
|  | Liquid stools | 1 | Vollebregt 2018 [12] |
|  | Constipation | 1 | Newton 2019 [8] |
|  | Inability to tell gas from stool | 1 | Dulai 2020 [5] |
|  | Loose stools | 1 | Dulai 2020 [5] |
|  | Odor to gas or stool | 1 | Dulai 2020 [5] |
| Pain and discomfort | Abdominal pain | 7 | Byron 2020 [1]; Calvet 2018 [2]; Carpio 2016 [3]; Chan 2017 [4]; Ghosh 2021 [14]; Hibi 2020 [11]; Newton 2019 [8] |
|  | Pain | 5 | Dubinsky 2021 [10]; Eluri 2018 [15]; Fourie 2018 [13]; Garcia-Alanis 2021 [6]; Hashash 2018 [7] |
|  | Abdominal cramping | 2 | Dulai 2020 [5]; Newton 2019 [8] |
|  | Joint pain | 1 | Dulai 2020 [5] |
|  | Back pain | 1 | Dulai 2020 [5] |
|  | Headache | 1 | Dulai 2020 [5] |
|  | Discomfort | 1 | Kim 2017 [16] |
| Energy-related | Fatigue | 8 | Calvet 2018 [2]; Carpio 2016 [3]; Chan 2017 [4]; Dubinsky 2021 [10] Dulai 2020 [5]; Eluri 2018 [15]; Garcia-Alanis 2021 [6]; Hashash 2018 [7] |
|  | Sleep disturbance | 6 | Dulai 2020 [5]; Eluri 2018 [15]; Hashash 2018 [7]; Lopez-Sanroman 2017 [17]; Newton 2019 [8]; Rapport 2019 [9] |
|  | Tiredness | 5 | Carpio 2016 [3]; Dulai 2020 [5]; Kim 2017 [16]; Newton 2019 [8]; Rapport 2019 [9] |
|  | Low energy | 3 | Dulai 2020 [5]; Fourie 2018 [13]; Rapport 2019 [9] |
|  | Weakness | 2 | Dulai 2020 [5] Kim 2017 [16] |
|  | Mental exhaustion | 1 | Dubinsky 2021 [10] |
|  | Feeling worn out | 1 | Rapport 2019 [9] |
| Nutrition | Weight loss | 5 | Byron 2020 [1]; Calvet 2018 [2] Dulai 2020 [5]; Newton 2019 [8] Rapport 2019 [9] |
|  | Dehydration | 2 | Dulai 2020 [5]; Newton 2019 [8] |
|  | Nutritional deficiencies | 1 | Chan 2017 [4] |
|  | Inability to eat without symptoms | 1 | Dubinsky 2021 [10] |
|  | Reduced appetite | 1 | Dulai 2020 [5] |
| Extraintestinal manifestations | Articular disease/arthralgia | 3 | Calvet 2018 [2]; Carpio 2016 [3]; Chan 2017 [4] |
|  | Dermatological issues | 2 | Calvet 2018 [2]; Dulai 2020 [5] |
|  | Ocular disease | 1 | Calvet 2018 [2] |
|  | Anemia | 1 | Dulai 2020 [5] |
|  | Irregular rapid heartbeat | 1 | Dulai 2020 [5] |
| Complications | Colectomy | 4 | Calvet 2018 [2]; Dubinsky 2021 [10]; Dulai 2020 [5]; Vollebregt 2018 [12] |
|  | Bowel resection | 2 | Dubinsky 2021 [10]; Vollebregt 2018 [12] |
|  | Perianal disease | 2 | Dulai 2020 [5]; Vollebregt 2018 [12] |
|  | Ileal pouch–anal anastomosis | 1 | Vollebregt 2018 [12] |
|  | Fistula | 1 | Vollebregt 2018 [12] |
|  | Abscess | 1 | Vollebregt 2018 [12] |
|  | Colorectal cancer | 1 | Dubinsky 2021 [10] |
| Flu-like symptoms | Sweating | 2 | Dulai 2020 [5]; Newton 2019 [8] |
|  | Light-headedness | 1 | Dulai 2020 [5] |
| Other symptoms | Psychological illness due to the bidirectional communication via the gut–brain axis | 1 | Barberio 2021 [18] |
|  | Somatization | 1 | Barberio 2021 [18] |

UC, ulcerative colitis.

**Supplemental Table S7**. UC proximal impact concepts and dimensions

| Dimension | Proximal impact concept | Number of studies reporting concept | Citation |
| --- | --- | --- | --- |
| Activities of daily living | Inability to conduct daily activities | 7 | Carpio 2016 [3]; Dubinsky 2021 [10]; Dulai 2020 [5]; Hibi 2020 [11]; Lopez-Sanroman 2017 [17]; Newton 2019 [8]; Rapport 2019 [9] |
|  | Need to be near toilet/ amount of time on toilet | 3 | Dubinsky 2021 [10]; Dulai 2020 [5]; Rapport 2019 [9] |
|  | Limitations to overall functioning | 2 | Chan 2017 [4]; Dulai 2020 [5] |
|  | Limitations to physical functioning | 1 | Dulai 2020 [5] |
|  | Difficulty concentrating | 1 | Dulai 2020 [5] |
|  | Housework/chores limitation | 1 | Dulai 2020 [5] |
|  | Unpredictability | 1 | Rapport 2019 [9] |
| Psychological | Anxiety | 8 | Chan 2017 [4]; Dubinsky 2021 [10]; Dulai 2020 [5]; Eluri 2018 [15]; Garcia-Alanis 2021 [6]; Kim 2017 [16]; Lopez-Sanroman 2017 [17]; Newton 2019 [8] |
|  | Depression | 7 | Chan 2017 [4]; Dubinsky 2021 [10]; Dulai 2020 [5]; Eluri 2018 [15]; Lopez-Sanroman 2017 [17]; Newton 2019 [8]; Vollebregt 2018 [12] |
|  | Embarrassment | 5 | Dulai 2020 [5]; Lopez-Sanroman 2017 [17]; Newton 2019 [8]; Rapport 2019 [9]; Vollebregt 2018 [12] |
|  | Worry/fear for future | 4 | Byron 2020 [1]; Dulai 2020 [5]; Lopez-Sanroman 2017 [17]; Newton 2019 [8] |
|  | Feeling isolated/ loneliness/ reluctance to disclose symptoms | 3 | Byron 2020 [1]; Hibi 2020 [11]; Rapport 2019 [9] |
|  | Fear of incontinence | 3 | Byron 2020 [1]; Dulai 2020 [5]; Rapport 2019 [9] |
|  | Frustration | 3 | Dulai 2020 [5]; Lopez-Sanroman 2017 [17]; Newton 2019 [8] |
|  | Anger | 3 | Byron 2020 [1]; Lopez-Sanroman 2017 [17]; Newton 2019 [8] |
|  | Stress | 2 | Dulai 2020 [5]; Kim 2017[16] |
|  | Reduced self-confidence/ low self-esteem | 2 | Lopez-Sanroman 2017 [17]; Newton 2019 [8] |
|  | Reduced quality of life | 2 | Barberio 2021 [18]; Ghosh 2021 [14] |
|  | Reduced general emotional health | 1 | Dulai 2020 [5] |
|  | Major mental disorder | 1 | Garcia-Alanis 2021 [6] |
|  | Substance use disorder | 1 | Garcia-Alanis 2021 [6] |
|  | Somatoform disorders | 1 | Garcia-Alanis 2021 [6] |
|  | Suicidal | 1 | Kim 2017 [16] |
|  | Feeling or reduced possibilities | 1 | Rapport 2019 [9] |
|  | Blame oneself for the illness | 1 | Byron 2020 [1] |
|  | Negative body image | 1 | Byron 2020 [1] |
|  | Eating disorder | 1 | Garcia-Alanis 2021 [6] |
|  | Irritability | 1 | Newton 2019 [8] |
|  | Lack of motivation | 1 | Lopez-Sanroman 2017 [17] |

UC, ulcerative colitis.

**Supplemental Table S8**. UC distal impact concepts and dimensions

| Dimension | Distal impact concept | Number of studies reporting concept | Citation |
| --- | --- | --- | --- |
| Lifestyle and activities | Inability to travel | 4 | Byron 2020 [1]; Dubinsky 2021 [10]; Dulai 2020 [5]; Rapport 2019 [9] |
|  | Having to plan around UC/ prepare for incontinence | 4 | Dubinsky 2021 [10]; Dulai 2020 [5]; Newton 2019 [8]; Rapport 2019 [9] |
|  | Reduced participation in leisure activities | 2 | Calvet 2018 [2]; Rapport 2019 [9] |
|  | Dietary changes | 2 | Dulai 2020 [5]; Newton 2019 [8] |
|  | Unable to leave house | 2 | Fourie 2018 [13]; Newton 2019 [8] |
|  | Negative effect of alcohol | 2 | Hashash 2018 [7]; Newton 2019 [8] |
|  | Eat less/avoid eating | 1 | Dulai 2020 [5] |
|  | Limitations to exercise/sport | 1 | Dulai 2020 [5] |
|  | Altered clothing choices | 1 | Dulai 2020 [5] |
|  | Affected lifestyle | 1 | Vollebregt 2018 [12] |
| Professional/ academic | Absence from work | 5 | Calvet 2018 [2]; Dubinsky 2021 [10]; Hibi 2020 [11]; Kim 2017 [16]; Rapport 2019 [9] |
|  | Unable to reach full potential | 3 | Calvet 2018 [2]; Kim 2017 [16]; Rapport 2019 [9] |
|  | Altered working hours | 3 | Byron 2020 [1]; Hibi 2020 [11]; Kim 2017 [16] |
|  | Impacted career choice | 3 | Barberio 2021 [18]; Calvet 2018 [2]; Hibi 2020 [11] |
|  | Retired early/quit job | 2 | Barberio 2021 [18]; Hibi 2020 [11] |
|  | Lack of support/understanding from employer and/or colleagues | 2 | Hibi 2020 [11]; Rapport 2019 [9] |
|  | Loss of productivity | 2 | Barberio 2021 [18]; Kim 2017 [16] |
|  | Absence from work when in remission | 1 | Dubinsky 2021 [10] |
|  | Non-disclosure for fear of repercussions | 1 | Dubinsky 2021 [10] |
|  | Negative effect on confidence at work | 1 | Dubinsky 2021 [10] |
|  | Difficulty scheduling medical appointments | 1 | Kim 2017 [16] |
|  | Felt stress due to time off | 1 | Kim 2017 [16] |
|  | Suffered stigma/discrimination | 1 | Kim 2017 [16] |
|  | Suffered illness effects at work | 1 | Newton 2019 [8] |
|  | Prefer to work alone | 1 | Byron 2020 [1] |
| Social functioning | Affected relationship with others (family/friends) | 5 | Calvet 2018 [2]; Dulai 2020 [5]; Kim 2017 [16]; Newton 2019 [8]; Rapport 2019 [9] |
|  | Reduced social participation | 3 | Byron 2020 [1]; Newton 2019 [8]; Rapport 2019 [9] |
|  | Unable to care for children | 2 | Calvet 2018 [2]; Rapport 2019 [9] |
|  | Relationship with partner affected | 1 | Dulai 2020 [5] |
|  | Limitations on social activities | 1 | Dulai 2020 [5] |
|  | Social isolation | 1 | Garcia-Alanis 2021 [6] |
|  | Prevented from making or keeping friends | 1 | Kim 2017 [16] |
|  | End of intimate relationship | 1 | Kim 2017 [16] |
| Sexual and reproductive | Influenced decision not to have children/number of children/ postponed having children | 3 | Calvet 2018 [2]; Dubinsky 2021 [10]; Fourie 2018 [13] |
|  | Fertility issues | 2 | Dubinsky 2021 [10]; Dulai 2020 [5] |
|  | Sexual function issues | 2 | Dulai 2020 [5]; Eluri 2018 [15] |
|  | Stopped treatment to have children | 1 | Dubinsky 2021 [10] |
|  | Postponed/ended/ avoided romantic relationships | 1 | Dubinsky 2021 [10] |
|  | Concerned about passing UC on to offspring/ decided to adopt | 1 | Dubinsky 2021 [10] |
| Other | Financial burden/ reduced income | 2 | Dulai 2020 [5]; Kim 2017 [16] |
|  | Financial burden of treatment | 1 | Kim 2017 [16] |
|  | Treatment burden | 1 | Dulai 2020 [5] |
|  | Frequent hospital admissions | 1 | Byron 2020 [1] |

UC, ulcerative colitis.


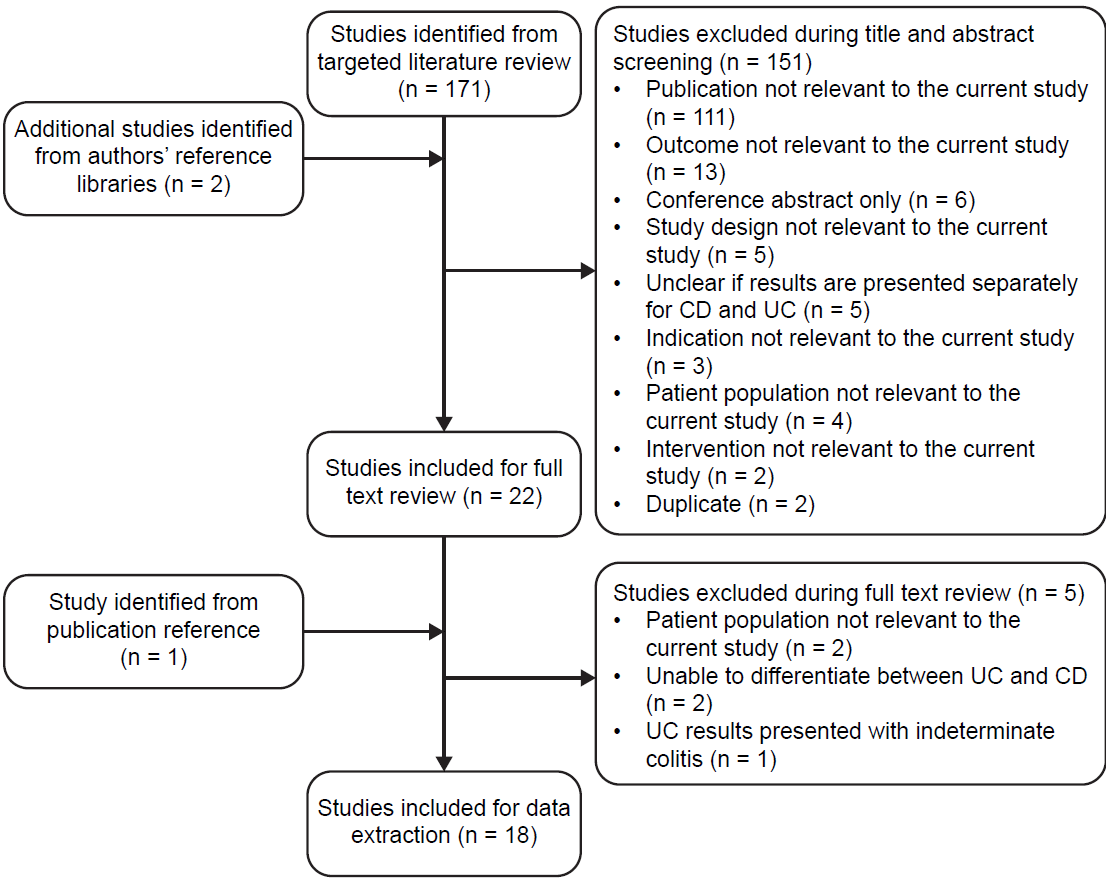


**Supplementary Figure S1.** Targeted literature review 1 flow chart.

CD, Crohn’s disease; UC, ulcerative colitis.


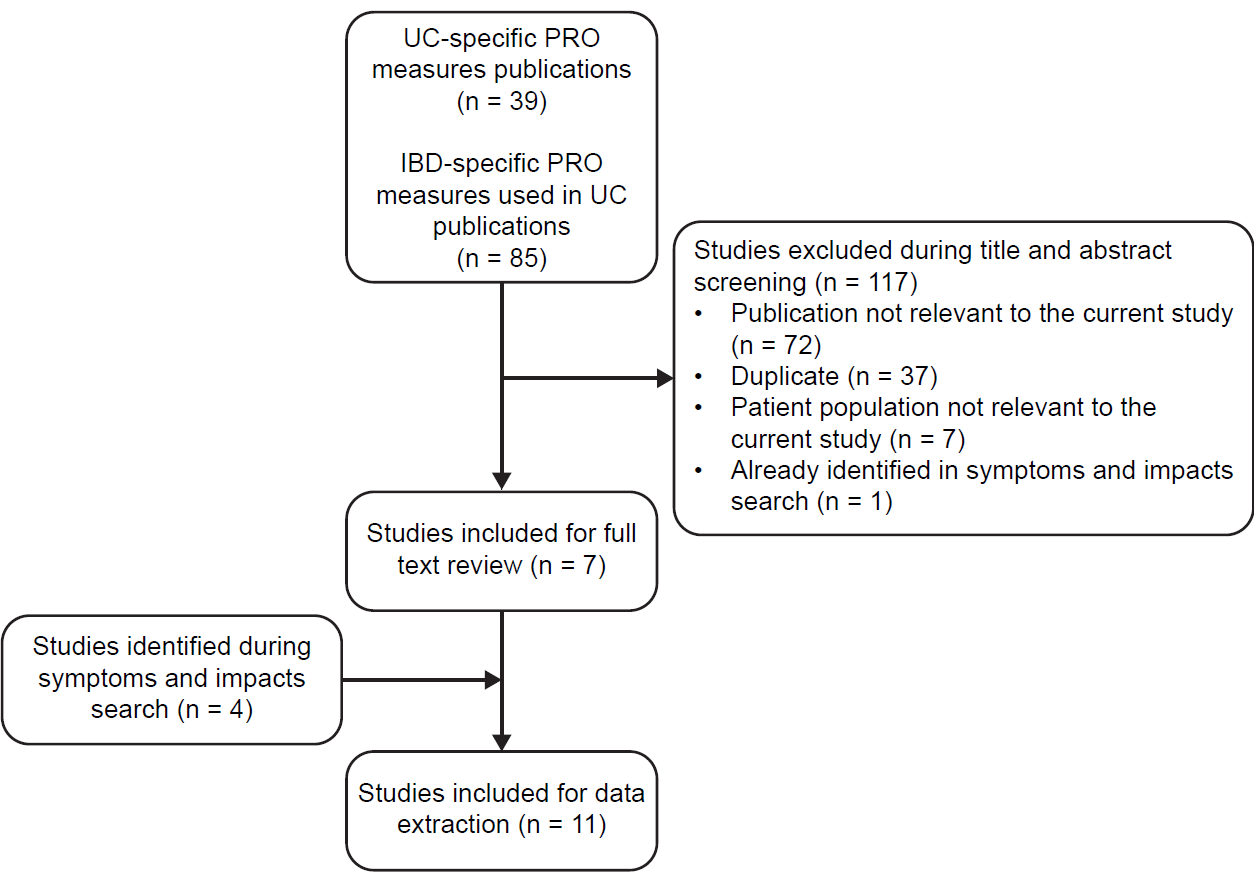


**Supplementary Figure S2.** Targeted literature review 2 flow chart.

IBD, inflammatory bowel disease; PRO, patient-reported outcome; UC, ulcerative colitis.

## References

1. Byron, C., Cornally, N., Burton, A., & Savage, E. (2020). Challenges of living with and managing inflammatory bowel disease: a meta-synthesis of patients' experiences. *J Clin Nurs*, 29(3–4), 305–319.

2. Calvet, X., Argüelles-Arias, F., López-Sanromán, A., Cea-Calvo, L., Juliá, B., de Santos, C. R., & Carpio, D. (2018). Patients' perceptions of the impact of ulcerative colitis on social and professional life: results from the UC-LIFE survey of outpatient clinics in Spain. *Patient Prefer Adherence*, 12, 1815–1823.

3. Carpio, D., López-Sanromán, A., Calvet, X., Romero, C., Cea-Calvo, L., Juliá, B., & Argüelles-Arias, F. (2016). Perception of disease burden and treatment satisfaction in patients with ulcerative colitis from outpatient clinics in Spain: UC-LIFE survey. *Eur J Gastroenterol Hepatol*, 28(9), 1056–1064.

4. Chan, W., Shim, H. H., Lim, M. S., Sawadjaan, F. L. B., Isaac, S. P., Chuah, S. W., Leong, R., & Kong, C. (2017). Symptoms of anxiety and depression are independently associated with inflammatory bowel disease-related disability. *Dig Liver Dis*, 49(12), 1314–1319.

5. Dulai, P. S., Jairath, V., Khanna, R., Ma, C., McCarrier, K. P., Martin, M. L., Parker, C. E., Morris, J., Feagan, B. G., & Sandborn, W. J. (2020). Development of the symptoms and impacts questionnaire for Crohn's disease and ulcerative colitis. *Aliment Pharmacol Ther*, 51(11), 1047–1066.

6. García-Alanís, M., Quiroz-Casian, L., Castañeda-González, H., Arguelles-Castro, P., Toapanta-Yanchapaxi, L., Chiquete-Anaya, E., Sarmiento-Aguilar, A., Bozada-Gutiérrez, K., & Yamamoto-Furusho, J. K. (2021). Prevalence of mental disorder and impact on quality of life in inflammatory bowel disease. *Gastroenterol Hepatol*, 44(3), 206–213.

7. Hashash, J. G., Ramos-Rivers, C., Youk, A., Chiu, W. K., Duff, K., Regueiro, M., Binion, D. G., Koutroubakis, I., Vachon, A., Benhayon, D., Dunn, M. A., & Szigethy, E. M. (2018). Quality of sleep and coexistent psychopathology have significant impact on fatigue burden in patients with inflammatory bowel disease. *J Clin Gastroenterol*, 52(5), 423–430.

8. Newton, L., Randall, J. A., Hunter, T., Keith, S., Symonds, T., Secrest, R. J., Komocsar, W. J., Curtis, S. E., Abetz-Webb, L., Kappelman, M., & Naegeli, A. N. (2019). A qualitative study exploring the health-related quality of life and symptomatic experiences of adults and adolescents with ulcerative colitis. *J Patient Rep Outcomes*, 3(1), 66.

9. Rapport, F., Clement, C., Seagrove, A. C., Alrubaiy, L., Hutchings, H. A., & Williams, J. G. (2019). Patient views about the impact of ulcerative colitis and its management with drug treatment and surgery: a nested qualitative study within the CONSTRUCT trial. *BMC Gastroenterol*, 19(1), 166.

10. Dubinsky, M. C., Watanabe, K., Molander, P., Peyrin-Biroulet, L., Rubin, M., Melmed, G. Y., Deuring, J. J., Woolcott, J., Cappelleri, J. C., Steinberg, K., & Connor, S. (2021). Ulcerative colitis narrative global survey findings: the impact of living with ulcerative colitis – patients' and physicians' view. *Inflamm Bowel Dis*, 27(11), 1747–1755.

11. Hibi, T., Ishibashi, T., Ikenoue, Y., Yoshihara, R., Nihei, A., & Kobayashi, T. (2020). Ulcerative colitis: disease burden, impact on daily life, and reluctance to consult medical professionals: results from a Japanese internet survey. *Inflamm Intest Dis*, 5(1), 27–35.

12. Vollebregt, P. F., van Bodegraven, A. A., Markus-de Kwaadsteniet, T. M. L., van der Horst, D., & Felt-Bersma, R. J. F. (2018). Impacts of perianal disease and faecal incontinence on quality of life and employment in 1092 patients with inflammatory bowel disease. *Aliment Pharmacol Ther*, 47(9), 1253–1260.

13. Fourie, S., Jackson, D., & Aveyard, H. (2018). Living with inflammatory bowel disease: a review of qualitative research studies. *Int J Nurs Stud*, 87, 149–156.

14. Ghosh, S., Sanchez Gonzalez, Y., Zhou, W., Clark, R., Xie, W., Louis, E., Loftus, E. V., Panes, J., & Danese, S. (2021). Upadacitinib treatment improves symptoms of bowel urgency and abdominal pain, and correlates with quality of life improvements in patients with moderate to severe ulcerative colitis. *J Crohns Colitis*, 15(12), 2022–2030.

15. Eluri, S., Cross, R. K., Martin, C., Weinfurt, K. P., Flynn, K. E., Long, M. D., Chen, W., Anton, K., Sandler, R. S., & Kappelman, M. D. (2018). Inflammatory bowel diseases can adversely impact domains of sexual function such as satisfaction with sex life. *Dig Dis Sci*, 63(6), 1572–1582.

16. Kim, Y. S., Jung, S. A., Lee, K. M., Park, S. J., Kim, T. O., Choi, C. H., Kim, H. G., Moon, W., Moon, C. M., Song, H. K., Na, S. Y., & Yang, S. K. (2017). Impact of inflammatory bowel disease on daily life: an online survey by the Korean Association for the Study of Intestinal Diseases. *Intest Res*, 15(3), 338–344.

17. López-Sanromán, A., Carpio, D., Calvet, X., Romero, C., Cea-Calvo, L., Juliá, B., & Argüelles-Arias, F. (2017). Perceived emotional and psychological impact of ulcerative colitis on outpatients in Spain: UC-LIFE survey. *Dig Dis Sci*, 62(1), 207–216.

18. Barberio, B., Zamani, M., Black, C. J., Savarino, E. V., & Ford, A. C. (2021). Prevalence of symptoms of anxiety and depression in patients with inflammatory bowel disease: a systematic review and meta-analysis. *Lancet Gastroenterol Hepatol*, 6(5), 359–370.
